# Dengue amid COVID-19 pandemic

**DOI:** 10.1371/journal.pgph.0001558

**Published:** 2023-02-06

**Authors:** Auchara Tangsathapornpong, Usa Thisyakorn

**Affiliations:** 1 Division of Pediatric Infectious Disease, Department of Pediatrics, Faculty of Medicine, Thammasat University, Pathumthani, Thailand; 2 Tropical Medicine Cluster, Chulalongkorn University, Bangkok, Thailand; University of Embu, KENYA

## Abstract

The increasing in dengue cases nowadays is a global threat concern. Fifty per cent of the world’s population is vulnerable to dengue infection with Asia contributing over two-thirds of the global burden. The double trouble of Coronavirus disease 2019 (COVID-19) arising from novel severe respiratory syndrome coronavirus (SARS-CoV-2) and dengue virus is a major challenge, particularly in developing countries due to overburdened public health systems and economic constraints including the ability to diagnose. The objective of this study was to analyze the prevalence of dengue in Thailand during the outbreak of COVID-19. We studied data on dengue cases reported at epidemiological information centers, the Bureau of Epidemiology, and the Ministry of Public Health, Thailand during 2019 to 2021. Patients can be observed across all age groups, particularly adolescents and adults. Dengue was seen year-round, with highest incidence in the rainy seasons between June and September. Total number of cases was markedly declined by nearly 93 percentage from 2019 to 2011. Taken together, Thailand is still at risk of spreading of dengue in the midst of COVID-19 pandemic. Continuous status updates on dengue patients in Thailand should be incorporated into global health advisory on preventive measures before travelling.

## Introduction

Dengue is an infectious, mosquito-borne, communicable viral disease stemming from four genetically related dengue virus serotypes. It is a major global public health threat affecting more than 120 countries. There were 5.2 million dengue cases reported in 2019. Fifty per cent of the world’s population is vulnerable to dengue infection with Asia contributing over two-thirds of the global burden. Since most dengue cases are asymptomatic and therefore difficult to detect, the nature of dengue infection can hamper surveillance activities [1]. Dengue is among the leading causes of hospitalization, which adds further pressure on already limited medical resources, together with adverse economic and social implications, especially in countries prone to dengue outbreaks. The disease is more common in the rainy season with all age groups affected [1, 2]. Dengue prevention and control depends primarily on effective tenable vector control [1]. Although limited efficacy of dengue vaccines, it is now used in a number of endemic countries [2–4]. Regarding the Pediatric Infectious Disease Society of Thailand, tetravalent dengue vaccine (Dengvaxia^TM^, CYD-TDV), became commercially available in 2016 and it is recommended at 6–45 year of age by using 3-dose series at 0, 6, and 12 months [5]. However, CYD-TDV vaccine has efficacy with a 65% rate of protection against dengue infection [6, 7].

The epidemiology of dengue in the Asia Pacific region is shifting in terms of its human host, the dengue virus, the vector bionomics, as well as the environment [8–10]. Many Asian countries have reported an epidemic change from dengue predominantly affecting children to impacting adolescents and young adults while the virulence and genotype of the virus determine the dengue disease severity [8–10]. The *Aedes* mosquito now survives longer and is found throughout cities and the countryside. The symptoms associated with dengue disease range from undifferentiated fever to severe and fatal infection related to plasma leakage, severe bleeding or organ impairment [8–10]. Prompt awareness of warning signs, plasma leakage, circulatory collapse, abnormal bleeding, respiratory distress and other complications would undoubtedly lower mortality rates in patients with dengue diseases. Adapting effectively to changing dengue epidemiology will lead to stronger operational policy for dengue control and more robust vaccine application strategies going forward [8–10].

In Thailand, dengue cases have been increasing annually from an average 69,000 cases per year from 1985 to 1999 to 91,650 cases per year from 2009 to 2015 [2, 11]. The highest incidence occurred in 2013 with approximately 154,000 and 156 deaths (241.03 cases per 100,000 populations) [11, 12]. Recent studies have reported economic burden of Dengue in Thailand was the third-highest ranked disease in South-East Asia Region [11, 12].

In a year of 2019, the Coronavirus disease 2019 (COVID-19) pandemic caused by novel severe respiratory syndrome coronavirus (SARS-CoV-2) has highlighted the vulnerability of health systems worldwide to diseases with already high rates of morbidity and/or mortality, including dengue infection. Interestingly, it was reported the total number of ‘active’ COVID-19 cases was markedly decreased, for instance, as of 2021 dropping by ~ 73% from September to December [13] after Thailand’s regulation of COVID-19 vaccines of second or third for a booster dose as recommended by WHO guideline [14]. This represents a significant reduction in the burden of hospitals.

The double trouble of COVID-19 arising from SARS-CoV-2 and dengue virus is a severe problem particularly in developing countries in aspect of overburdened public health infrastructure, as well as economic constraints, including diagnostic capability. Healthcare services utilization at the inpatient, outpatient, and emergency department has responded to resource limitation which leads to insufficient levels of healthcare in a timely manner [15, 16]. Additionally, the pandemic has created an over workload burden for healthcare professionals/providers, which may result in exhaustion and mental distress [15, 16].

Therefore, the purpose of this study was to analyze the prevalence of dengue in Thailand during the outbreak of COVID-19.

## Materials and methods

We performed an analysis of dengue patient quantitative data reported to the epidemiological information center, Bureau of Epidemiology, Ministry of Public Health, Thailand. The data was collected from January 2019 to December 2021, including information on age, reporting area (province), reporting month and outcomes in terms of mortality [8]. The diagnosis of all patients adhered to the 1997 World Health Organization (WHO) dengue classification as Table 1 [9]. This study was approved by the Ethics Review Committee of the Faculty of Medicine, Thammasat University (Number of certificate approval: 136/2022). As this study was a retrospective, informed consent was waived.

**Table 1:** Definition for dengue diseases; adapted from reference [9].

Clinical criteria	Signs and symptoms
1. Dengue Fever (DF)	• Acute fever with more than one of the following: • Pain behind the eyes • Headache • Rash • Arthralgia • Myalgia • Hemorrhagic manifestations • Leukopenia
2. Dengue Hemorrhagic Fever (DHF)	Must include all of the following: • Acute fever (duration 2–7 days) • Bleeding/hemorrhagic manifestations: petechiae, ecchymosis, positive tourniquet test or bleeding from the mucous membranes, gastrointestinal tract (melena/hematemesis) or bleeding in other sites • Thrombocytopenia (platelets count less than 100,000 per mm^3^) • Evidence of plasma leakage caused by increased vascular permeability
3. Dengue Shock Syndrome (DSS)	• DHF with narrow pulse pressure (>20 mmHg) or hypotension, add one of the following: • Weak and rapid pulse • Restlessness, cold/clammy skin
Laboratory criteria:	Positive dengue serology or occurrence at the similar location and time as other confirmed patients.

The Statistical Software for Excel 2022, were used for all statistical data analysis. Demographic data and outcomes were analyzed using descriptive statistics. The numerical data were presented as the distribution grouped frequency of a variable by histogram graph. The relationship between two parameters was illustrated as linear graph.

## Results

**Fig 1:**
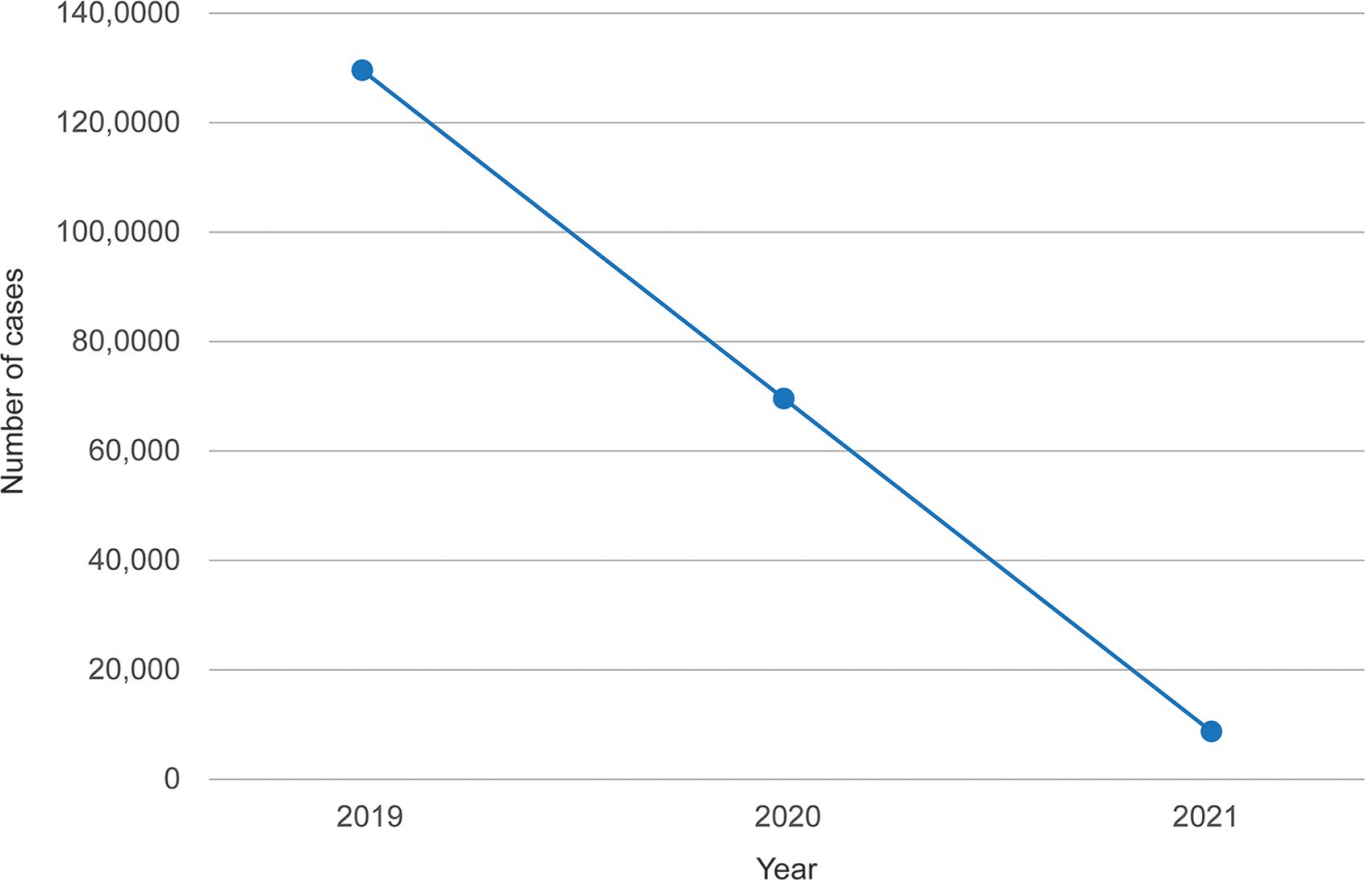
Number of dengue patients in Thailand between 2019 and 2021.

Dengue patients in Thailand during the study period of 2019 to 2021 are shown in Fig 1; a total of 213,632 dengue patients were reported in Thailand, of which 131,157 occurred in 2019, then 72,519 in 2020 and 9,956 in 2021, respectively (Fig 1).

**Fig 2:**
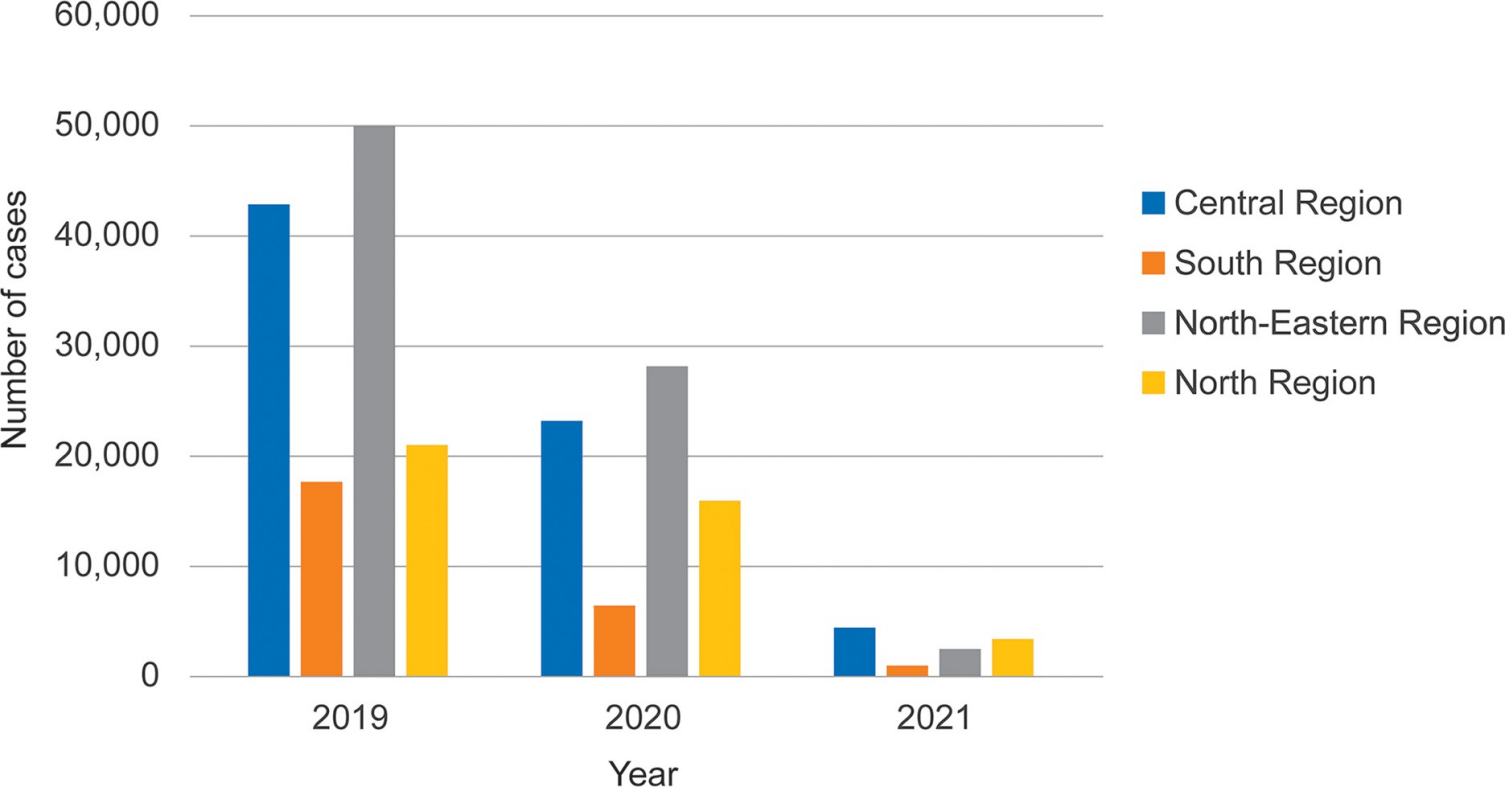
Distribution of dengue patients from different regions in Thailand from 2019 to 2021.

**Fig 3:**
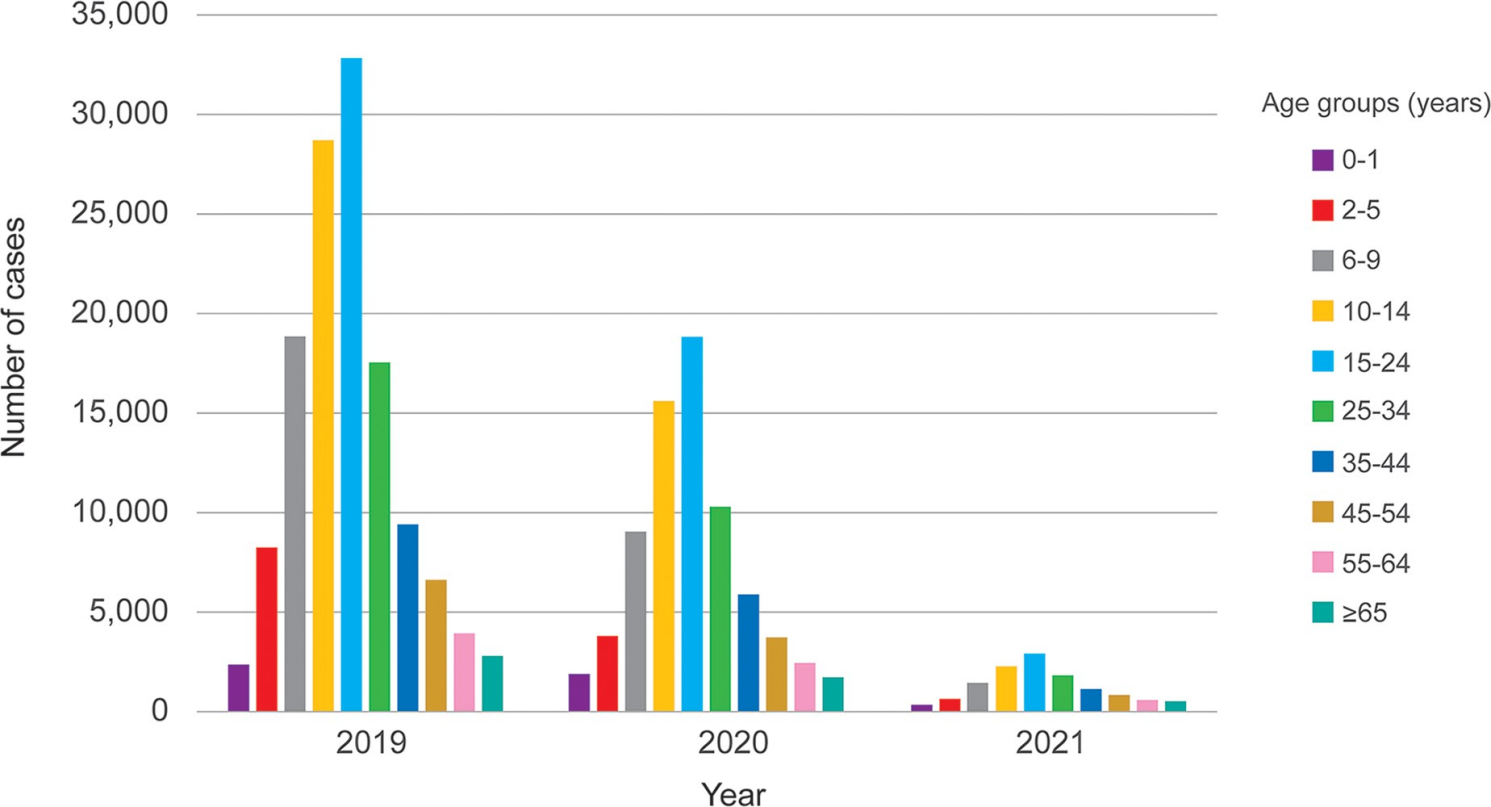
Age distribution of dengue patients in Thailand from 2019 to 2021.

**Fig 4:**
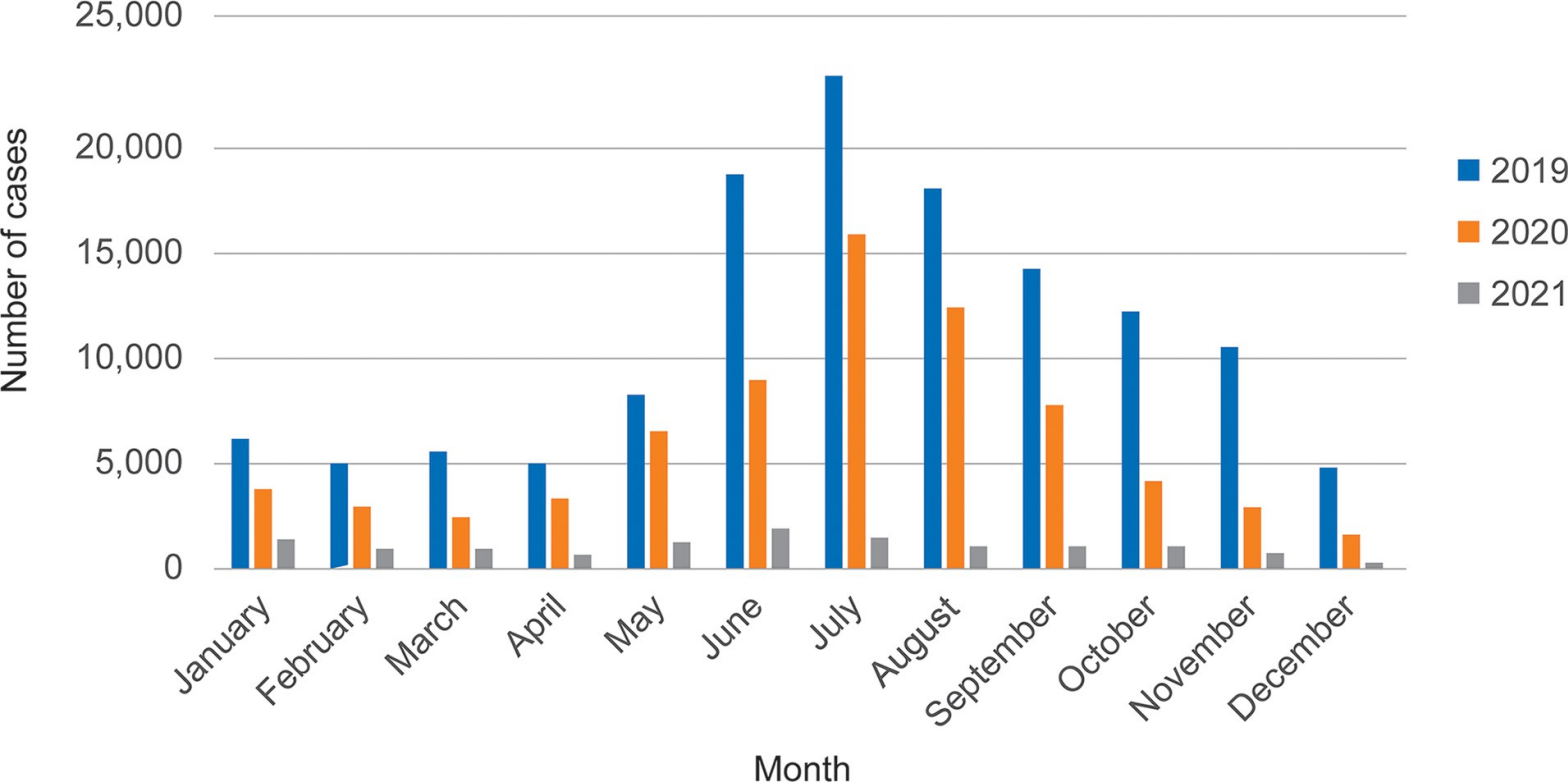
Seasonal distribution of dengue patients in Thailand from 2019 to 2021.

Patients can be seen across all age groups, more so in adolescents and adults, with children (<15 years) accounting for 92,097 cases (43.1%), and adults numbering 121,535 cases (56.9%). The highest amount of dengue cases was found in those aged 15–24 years (25.2%), followed by 10–14 years (21.5%), 6–9 years (13.7%), 25–34 years (13.5%), 35–44 years (7.5%), 2–5 years (6.1%), 45–54 years (4.9%), 55–64 years (3.5%), ≥ 65 years (2.3%) and < 1 years (1.8%), respectively (Fig 2). Dengue patients were found in all regions of Thailand (Fig 3). The disease was seen throughout the year especially the rainy season from June to September (Fig 4). The overall case fatality rate from dengue was 0.09% (*n* = 189) with 0.1% in 2019, 0.07% in 2020, and 0.06% in 2021, respectively.

## Discussion

The COVID-19 pandemic has greatly impacted healthcare systems worldwide to diseases with high morbidity and/or mortality, including dengue. The double trouble of COVID-19 dengue virus notably impact on medical care management. During COVID-19 pandemic in Thailand in a period of 2019–2021, our study provides epidemiological data showing dengue diseases remain amid the outbreak of COVID-19 and can be observed across all age groups, especially in adolescents and adults, with no differences in gender. The disease was observed throughout the year particularly the rainy season from May to October. The number of cases was markedly decreased from 2019 to 2021.

Healthcare providers are now faced with a considerable challenge in dealing with dengue virus, mainly in areas unfamiliar with it due to its transmission to humans via the vector *Aedes aegypti* mosquito. An outbreak has repercussions on all healthcare providers since it is mandatory to disclose dengue cases to provincial or local health authorities. Considering the global nature of dengue virus, healthcare providers should cooperate beyond national borders and medical disciplines in order to improve patients’ understanding of dengue manifestations and the mosquito vector.

The impact of COVID-19 and dengue has doubled in difficulty, particularly in developing countries due to the overburdened public health system, economic barriers and limited diagnostic capability [17, 18]. Misdiagnosis or co-infection with COVID-19 and dengue is a great concern [19–22]. COVID-19 and dengue have overlapping clinical manifestations and similar laboratory features, leading to possible misdiagnosis. There have also been reports of co-infection between COVID-19 and dengue in Brazil, Singapore, Indonesia and Thailand [23–27]. In South America and Southeast Asia, where both viruses are in co-circulation, 22% of COVID-19 patients are estimated to be falsely dengue positive which will jeopardize SARS-CoV-2 virus containment [28]. Therefore, it should be cautiously interpreted the laboratory results.

To distinguish between the diagnostic tests for SARS-CoV-2 and Dengue, a simple, affordable, and accurate rapid methods were described in previous studies [27–30]. The diagnostics for SARS-CoV-2 infection during pandemic is currently performed by detection of SARS-CoV-2 RNA using a reverse transcriptase-polymerase chain reaction (RT-PCR) test from oral or nasopharyngeal swabs [31, 32]. With regard for diagnosis dengue virus infection as established by the World Health Organization [9], clinical manifestations with laboratory including leukopenia, thrombocytopenia or evidence of plasma leakage are crucial for diagnosis. A positive dengue serology/NS1 Ag-based test or occurrence at the similar location and time as other confirmed patients are concurrently considered. Even though, updated meta-analysis study suggested NS1 Ag-based test was a good diagnosis method for dengue with a high specificity but limited in some resource-limited settings [33].

In line with dengue treatment during high peak time of Covid-19, treatment options are similar to those in before COVID-19 pandemic according to WHO guidelines [34]. Current treatments are supportive and limitation of the complications and severity of symptoms. However, all Dengue patients will be screened for the risk of COVID-19 infection by questionnaire. Severe and critical Dengue patient had undergone COVID-19 determining of active COVID-19 infection by RT-PCR test through oral and nasopharyngeal prior to hospitalization. On the other hand, pre- hospital nasal or saliva rapid antigen test kit screening has been widely recommended [35]. The patient who has a positive COVID 19 test results was separately admitted to the isolated Covid-19 ward in the hospital from negative COVID-19 infected Dengue patients. Nevertheless, it is safe to rescreen only high-risk hospitalized patients.

Another challenge that must be faced during the pandemic is the possibility of ongoing vector control. Several countries in South America and Southeast Asia reported an increase in dengue cases during the lockdown. Cessation of vector control measures due to diversion of resources during the pandemic could be responsible for an increasing in dengue [36]. However, the pandemic has endangered dengue control, especially in high-risk countries, which should proactively reinforce prevention and control efforts. During the pandemic, the need for dengue preparedness and strengthening public health infrastructure must be emphasized.

Dengue vaccination is an important public health measures and requires ongoing support through the participation of healthcare workers, policy makers, public and media, which will play an important role in enhancing public confidence and maintaining vaccination [37].

WHO has proposed a worldwide strategy to prevent and control dengue infection (2021–2030) to curtail preventable dengue deaths to zero and to lower dengue incidence by 25% [38]. To achieve this goal, dengue must be recognized as a global threat. Collaborative efforts are needed to strengthen the readiness, prevention and control of dengue. There is an urgent need to work together to strengthen the readiness, prevention and control. It is a multi-sector collaboration between the ministries of health, education and environment, the community and the private sector to effectively control dengue fever around the world.

Thus, the COVID-19 and dengue epidemic occurring simultaneously is a worrying concern. It is necessary that health care providers and governmental health authorities are need to be aware of the risks associated with co-existing such diseases and to developed an epidemiological surveillance and action plan to alleviate pressure on health facilities.

There are indications that in Thailand, dengue has already morphed into persistent endemic transmission. Dengue cases were noted throughout the year from 2019 to 2021, with an uptick from June to September, considered to be the rainy season. Even though all age groups were affected, dengue cases were considerably/predominantly higher in adolescents and adults. However, the number of reported dengue patients dropped during 2019 to 2021, which corresponded with higher numbers of COVID-19 patients from the delta strain. With this particular finding, it is feasible social distancing during the COVID-19 pandemic enacted by the government could reduce a transmission of dengue virus. Interestingly, all regions of Thailand were dramatically decreased of dengue cases implying the effectiveness of the government regulation. On the other hand, resource limitation and exhausted healthcare services during the COVID-19 outbreak should not be excluded. This was a limitation of our study.

## Conclusion

Highlight of our findings rests on the fact that we showed the epidemiologic data to underpin dengue diseases remain in Thailand amid the COVID-19 pandemic. Patients are observed in all age groups, particularly adolescents and adults. The highest incidence of dengue is typically found in rainy seasons between June and September. Continuous status updates on dengue diseases in Thailand should be incorporated into global health advisory on preventive measures before travelling.
